# Proteomic Analysis Revealed that DVL, a Lectin Purified from *Dioclea violacea* Seeds, Induced a Change in the Protein Profile of *Candida albicans*

**DOI:** 10.1021/acsomega.5c04868

**Published:** 2025-08-29

**Authors:** Romério R. S. Silva, Maria H. C. Santos, Ana L. E. Santos, Cleverson D. T. Freitas, Rômulo F. Carneiro, Celso S. Nagano, Felipe P. Mesquita, Pedro F. N. Souza, Claudener S. Teixeira

**Affiliations:** † Department of Biochemistry and Molecular Biology, 28121 Federal University of Ceará, Fortaleza 60451-970, CE, Brazil; ‡ Medical School, Federal University of Cariri, Barbalha, Ceará 63048-080, Brazil; § Department of Fisheries Engineering, Federal University of Ceará (UFC), Fortaleza, CE 60430-275, Brazil; ∥ Laboratory of Pharmacogenetics, Center for Drug Research and Development (NPDM), Department of Physiology and Pharmacology, Federal University of Ceará, Fortaleza 60430-275, CE, Brazil; ⊥ Laboratory of Bioinformatics Applied to Human Health, Center for Drug Research and Development (NPDM), Federal University of Ceará, Fortaleza 60020-181, Brazil; # National Institute of Science and Technology in Human Pathogenic Fungi (FunVir), Fortaleza 60 356-150, Brazil; ¶ Visiting Researcher at the Cearense Foundation to Support Scientific and Technological Development, Fortaleza, CE 60 356-150, Brazil

## Abstract

*Candida albicans* is an important opportunistic fungal pathogen, and its resistance to conventional treatments poses a substantial challenge. Previous research by our group demonstrated that the anticandidal activity of *Dioclea violacea* seed lectin (DVL) involves multiple mechanisms of action. Our current objective is to analyze changes in the proteome of *C. albicans* cells after treatment with the DVL lectin. The proteomic analysis corroborated the previously observed mechanisms with greater specificity, encompassing processes such as cell wall integrity, expression of transport proteins, proteins related to metabolism and energy, DNA repair proteins, and proteins related to defense and stress, and downregulated cell cycle proteins affecting cell viability. Our findings provide novel insights into *C. albicans* response to DVL lectin, emphasizing the intricate cellular mechanisms underlying stress adaptation. These results provided new insight into the mechanisms of action of DVL against *C. albicans*. They may facilitate the development of more effective and innovative antifungal therapies by providing a comprehensive understanding of fungal pathogenesis.

## Graphical abstract

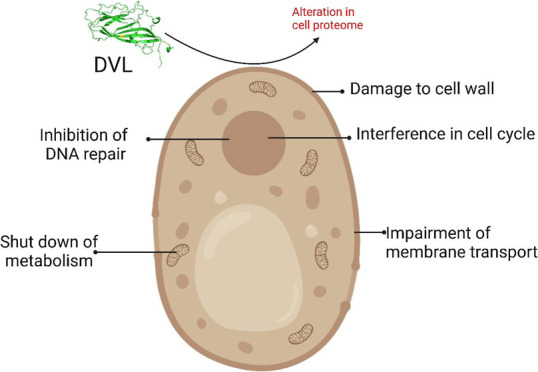

## 1 Introduction

Fungal infections pose a growing global health threat due to limited options and the emergence of antifungal resistance. This resistance to conventional antifungal agents presents a significant challenge, hindering the medical community’s efforts to develop effective therapies.^1,2^ *Candida albicans* is a primary opportunistic fungal pathogen in humans and a formidable challenge due to its ability to cause severe infections, particularly in the elderly and immunocompromised populations.^3–5^ The urgent need for novel therapeutic strategies to combat this resistant pathogen is evident.

Plant lectins, known for their antifungal properties, offer promising alternatives to conventional antifungals. These proteins bind reversibly to carbohydrates, a key factor in their anti-*candida* activity.^6–8^ Our previous study^9^ demonstrated that the lectin extracted from *Dioclea violacea* seeds exhibited significant anti-*candida* activity against *C. albicans* through multiple mechanisms of action, including inhibition of ergosterol biosynthesis and damage to the cell membrane and wall. However, this activity’s molecular mechanisms have not yet been fully elucidated.

Although studies have explored the biological effects of plant lectins on anti-*candida* activity, there is a significant lack of investigations based on proteomic studies to understand this antifungal action at the molecular level. This gap limits the understanding of the specific cellular targets of these proteins and their potential therapeutic applications.

Using proteomic analysis, we aim to elucidate how *C. albicans* cells respond to DVL lectin exposure by identifying key proteins and molecular pathways. Proteomics offers a robust approach to profiling cellular proteins, potentially uncovering novel antimicrobial biomarkers and therapeutic targets, significantly advancing the combat against resistant fungal infections.^10,11^ In addition, plays a crucial role in identifying pathogens, elucidating pathogenesis mechanisms, accurately diagnosing diseases, discovering potential antifungal drugs, and developing innovative therapeutic approaches to treat fungal infections.^12,13^ Proteomic techniques offer a powerful approach to comprehensively investigate the evolution of resistance in pathogenic fungi over time.^14^ Several studies have employed proteomics analysis to understand the mechanisms of action of antimicrobial proteins and peptides.^15–18^

Proteomic studies have characterized the *C. albicans* biofilm proteome, identifying key proteins associated with biofilm formation and resistance.^19,20^ Additionally, proteomic analyses have explored the mechanisms underlying *C. albicans* antifungal resistance, revealing potential therapeutic targets.^14^ While our previous research established the antifungal activity of DVL lectin against *C. albicans*,^9^ there is currently no published proteomic data on *C. albicans* responses to plant lectins.

Given these considerations, this study aims to investigate, through proteomic analysis, the changes in the protein profile of *C. albicans* in response to treatment with DVL lectin. Our goal is to elucidate the antifungal mechanisms involved, highlighting the metabolic pathways and modulated proteins, to contribute to understanding the potential of plant lectins as antifungal agents.

## 2 Material and Methods

### 2.1 Biological Material

The *D. violacea* seeds were harvested from plants in Vargem Grande city, Maranhão, Brazil, and registered in the National System for the Management of Genetic Heritage and Associated Traditional Knowledge with an ID: AF8E1DD. Fungal cells used in this study, *C. albicans* (ATCC 10231), were obtained from the Department of Biochemistry and Molecular Biology of the Federal University of Ceará (UFC).

### 2.2 Purification of DVL from *D. Violacea* Seeds

DVL was extracted and purified, as described by Silva et al.^9^ The purification was judged by SDS-PAGE.^21^ The chromatogram showed two peaks (Figure 1). The first peak (PI) represents the proteins that did not interact with the column. The second (PII) regards retained protein fraction (Figure 1A). The PII (lane L3 in SDS-PAGE) represents purified DVL showing three bands (Figure 1B), the first band corresponding to the α-chain (25 kDa), the second to the β-chain (16 kDa), and the third to the γ-chain (12 kDa).

**Figure 1:**
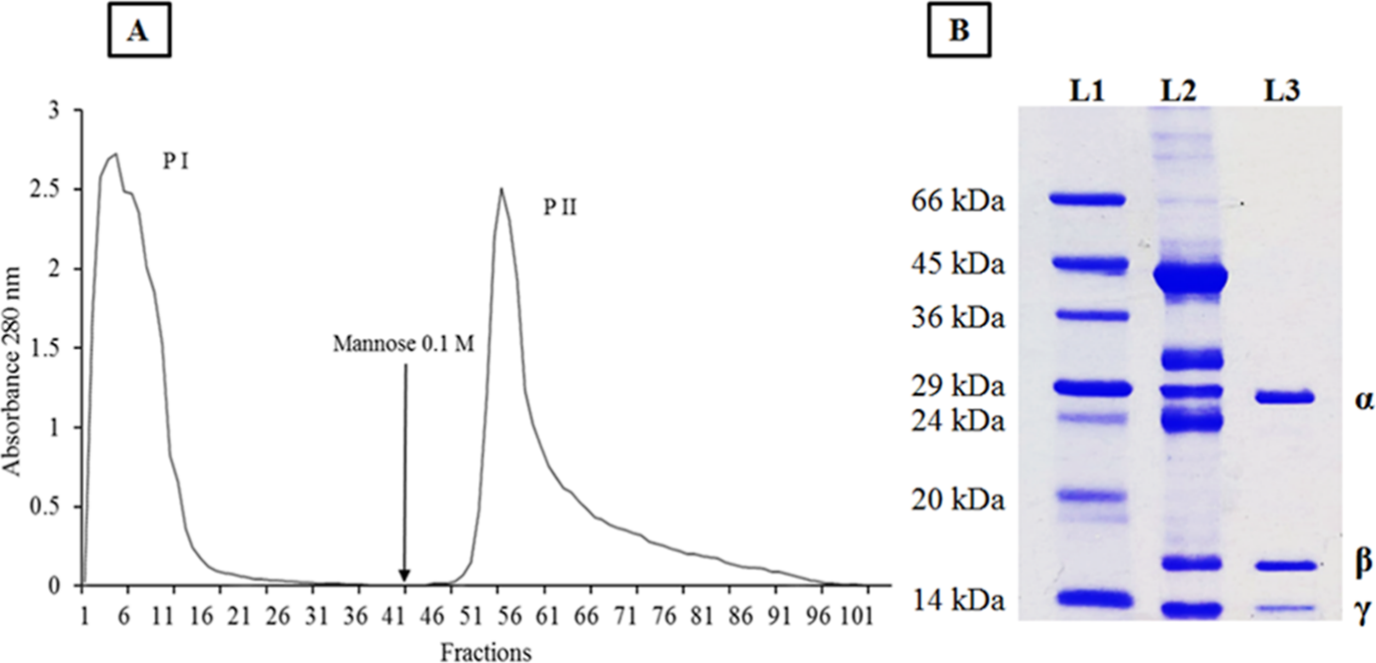
DVL purification by affinity chromatography. (A) Chromatogram profile of *D. violacea* in Sephadex-G-75 column. (B) SDS-PAGE is showing DVL after purification. L1: molecular weight standards (MW); L2: crude extract; L3: PII (purified DVL). Molecular weight marker: bovine serum albumin, 66 kDa; ovalbumin, 45 kDa; glyceraldehyde-3-phosphate dehydrogenase, 36 kDa; carbonic anhydrase, 29 kDa; trypsinogen, 24 kDa; trypsin inhibitor, 20 kDa; α-lactalbumin, 14 kDa; (DVL) α-chain, β-chain, and γ-chain of lectin.

### 2.3 Antifungal Activity

The antifungal potential of DVL against *C. albicans* was performed as previously defined in our published study.^9^ An aliquot (50 μL) of *C. albicans* suspensions (2 × 10^3^ CFU mL^–1^) in Sabouraud broth medium was incubated for 24 h at the concentration MIC_50_ 0.6 μM for at 37 °C, using polystyrene flat-bottom 96-well microtiter plates method described by the Clinical and Laboratory Standards Institute,^9^ and cell growth was measured using a microplate reader at 600 nm (Epoch, BioTek Instruments Inc., Winooski, VT, USA). The experiments were performed three times, with three replicates per treatment. Itraconazole (1000 μg mL^–1^) and 0.15 M NaCl were positive and negative controls, respectively.

### 2.4 Extraction of Proteins from *C. Albicans* Cells

The proteomic analysis was done using the control cells and cells treated with DVL. Protein extraction from *C. albicans* cells was carried out according to the protocol of Branco et al.^22^ To increase the yield of protein for proteomic analysis in the proteomic assays was used *C. albicans* suspensions at 2 × 10^6^ cfu mL^–1^. The samples were washed 3× with 50 mM sodium acetate pH 5.2 (extraction buffer) buffer removed the culture media and centrifuged at 12,000*g* for 15 min at 4 °C. After resuspension in 200 μL of extraction buffer, cells were frozen at −20 °C for 24 h, sonicated for 30 min to break the cell wall and plasma membrane, frozen for 24 h and sonicated again for 30 min centrifuged again and the supernatant collected, and assayed for Bradford reagent to quantify proteins^23^ using bovine serum albumin (BSA). In the end, the extracted proteins were used for proteomic analysis.

### 2.5 LC/MS–MS Mass Spectrometry Analysis

Before analysis, proteins were reduced with 10 mM DTT and incubated for 1 h at 37 °C to reduce the proteins, alkylated with 15 mM of iodoacetamide for 30 min, and finally digested for 16 h at 37 °C in the dark with gold trypsin (Promega, Madison, WI, USA) at a final concentration of 1:20 (w/w), as described by the manufacturers. After digestion, the samples were dried in a rapid vacuum (Eppendorf, Hamburg, Germany) for 3 h, resuspended in 0.1% formic acid, and analyzed by nano-HPLC coupled to an ESI-QUAD-TOF mass spectrometer.

### 2.6 Protein Identification

Protein identification was carried out according to Branco et al.^22^ The tandem mass spectra were exported as.pkl files and loaded into the MASCOT MS/MS ion search from MATRIX SCIENCE (https://www.matrixscience.com/cgi/search_form.pl?FORMVER=2&SEARCH=MIS, accessed October 20, 2023) against UP2311_*S_cerevisiae* (protein database) and UP219602_*F_oxysporum* (protein database). The search used the following parameters: the peptide charge was set to 2+, 3+, and 4+, variable Oxidation (O), and fixed modifications in Carbamidomethyl (C). The identified proteins were classified into 3 sets: (1) unique to the control for those identified only in the control samples, (2) unique to the cells treated with the DVL lectin for those identified only in the treated samples, and (3) DVL x Control overlapping proteins.

Proteins with a fold change value ≥1.5 (*p* < 0.05, Tukey’s test)^22^ were up-accumulated (increased abundance), and proteins with a fold change value ≤0.5 (*p* < 0.05, Tukey’s test) were down-accumulated (decreased abundance) and considered for comparisons. Proteins with a fold change value between 0.5 and 1.5 were considered unchanged.^22^ The corresponding FASTA file was downloaded for each sample. The blast2go program (https://www.blast2go.com/, accessed November 15, 2023) was used to categorize the proteins blocked by Gene Ontology (GO) annotation according to molecular function, biological activity, and subcellular location.

### 2.7 Statistical Analysis

The proteomic analysis was performed individually three times, and the values were expressed as the mean ± standard error. The data were submitted to ANOVA software, followed by the Tukey test. GraphPad Prism 5.01. (GraphPad Software Company, California, USA) was used to perform all graphics, with a significance of *p* < 0.05.

## 3 Results and Discussion

### 3.1 Overview

DVL is a d-glucose/mannose lectin with potent antifungal activity against *C. albicans*. The potency of DVL is highlighted when it is compared to other lectins, such as MaL, a lectin from *Machaerium acutifolium* seeds.^8^ For example, MaL presented an MIC_50_ against *C. albicans* 30 (18 μM) times higher than DVL (0.6 μM). Other lectins, such as *Canavalia rosea* and *C. ensiformes*,^7^ did present antifungal activity. Based on that, and as stated in our previous work,^9^ DVL against *C. albicans,* such as increased membrane permeabilization, pore formation, and damage to the cell wall. In addition, it induces overaccumulation of ROS and inhibits the biosynthesis of ergosterol in *C. albicans* cells.^9^ Here, proteomic analysis was employed to investigate the changes in the cellular proteomic profile caused by DVL on *C. albicans*. We employed LC-ESI-MS/MS to identify proteins in DVL-treated *C. albicans* cells compared to control cells. A total of 289 proteins were identified, including 139 from untreated cells (Table S1), 136 from DVL-treated cells (Table S2), and 14 common to both conditions (Table S1). Among these 14 proteins in overlap, we observed that two are increased in abundance after treatment with DVL with a fold-change >1.5 (*p* > 0.05 Tukey test). In contrast, only one decreases in abundance with a fold-change <0.5 (*p* > 0.05 Tukey test). The other 10 proteins showed no change in their accumulation.^24^

Gene Ontology analysis of the proteins shared between DVL-treated cells and the *C. albicans* control revealed that several biological processes, molecular functions, and cellular components were affected (Figure 2). The group with the greatest representation in biological processes was the long-chain fatty acid biosynthetic process, with a fold-enrichment higher than 40. This is an interesting result, because in our previous work^9^ we reported that DVL inhibited the ergosterol biosynthesis in *C. albicans* cells. Fungi typically alter membrane composition to adapt to and survive antifungal drug-induced cellular stress.^25^

**Figure 2:**
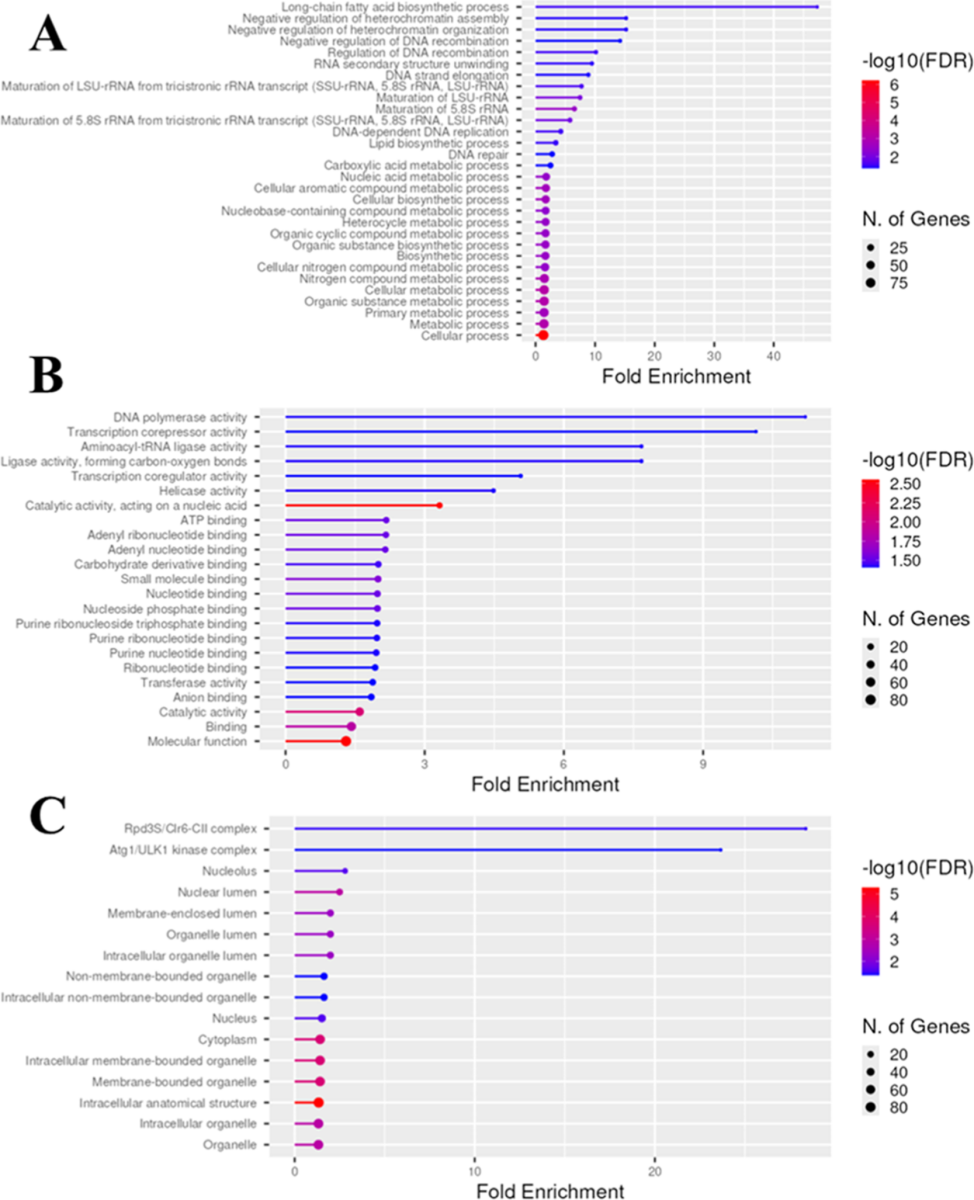
Gene ontology of proteins identified by proteomic analysis. (A) Biological process, (B) molecular function, and (C) cellular component classified based on gene ontology using the Uniprot database considering a false discovery rate (FDR) < 0.05.

The classification of proteins in biological^26^ processes and molecular function is diverse and covers all aspects regarding cell life (Figure 2). The gene ontology of proteins from DVL-treated cells revealed a different scenario (Figure 3). The abundant group present classified in control cells suffered a reduction in treated cells, suggesting the effect of DVL in *C. albicans* cells transferase (3%), gene regulation (2%), transmembrane transport (7%), amino acids metabolism (2%), and protein biosynthesis (7%). These results suggest which pathways are affected by DVL treatment and provide a clue about the mechanism employed by the lectin to affect *Candida* cells.

**Figure 3:**
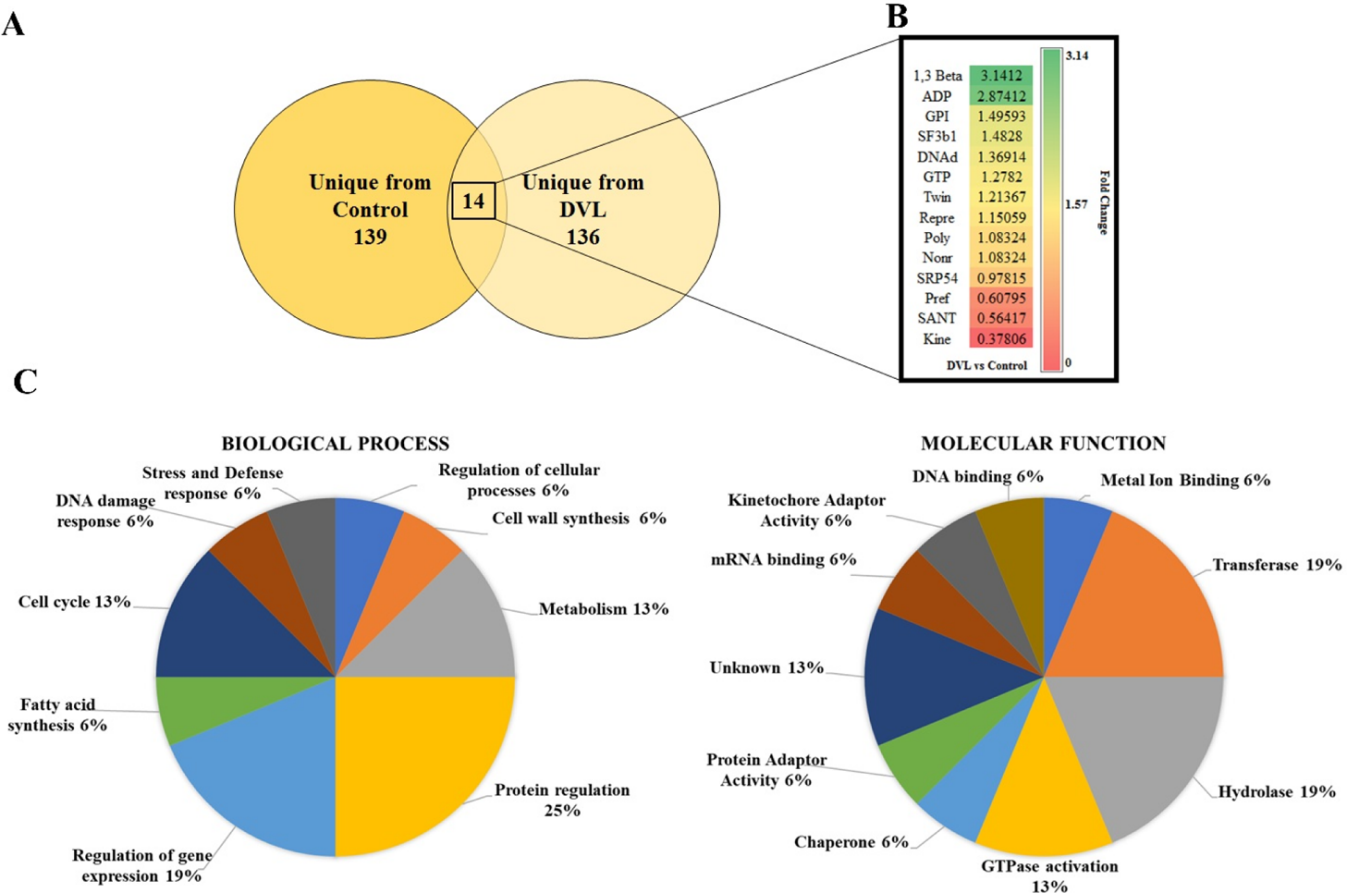
Differentially accumulated proteins in*C. albicans* cells treated with the lectin DVL. In (A), the Venn diagram highlights the differential distribution of proteins in single treated and untreated cells from each group and the overlapping protein found in both groups with differential accumulation. (B) Proteins are classified according to biological processes. (C) Proteins are classified according to molecular function. Statistical analysis by Tukey’s test did not indicate statistical significance among the proteins analyzed (*p* < 0.05).

### 3.2 Cell Wall Synthesis Proteins

In this group, only one protein was identified with an overlap in the treated and control cells. The protein 1,3- β -glucan synthase component GSC2 (1-3GSC2) had up-accumulation (3.1-fold) in DVL-treated cells compared to control cells (Table S1). 1-3GSC2 is an important enzyme involved in the biosynthesis of 1,3-β-glucan that composes the cell wall of fungi.^27,28^ In turn, the fungal cell wall plays a fundamental role in regulating cellular functions such as cell stability, permeability, and protection against stress.^29^ Under stress, when the fungal cell wall is damaged, it is necessary to repair it. At this point, 1-3GSC2 is necessary for cell wall turnover.^30^ Therefore, the increase in 1-3GSC2 DVL-treated *C. albicans* cells indicates damage in the cell wall and that cells are trying to recover from it. This result corroborates our previously published work.^9^ By employing scanning electron microscopy, it was shown that DVL induced severe damage to the morphology of *C. albicans* cells. Based on those results, we reasoned that the increase in abundance of 1-3GSC2 could be an adaptive response to reinforce the integrity of the cell wall and repair the damage caused by DVL (Figure 4). However, based on what was shown before, *C. albicans* cannot recover from this damage once the DVL-treated *C. albicans* cells present a loss of cytoplasmic content.^9^

**Figure 4:**
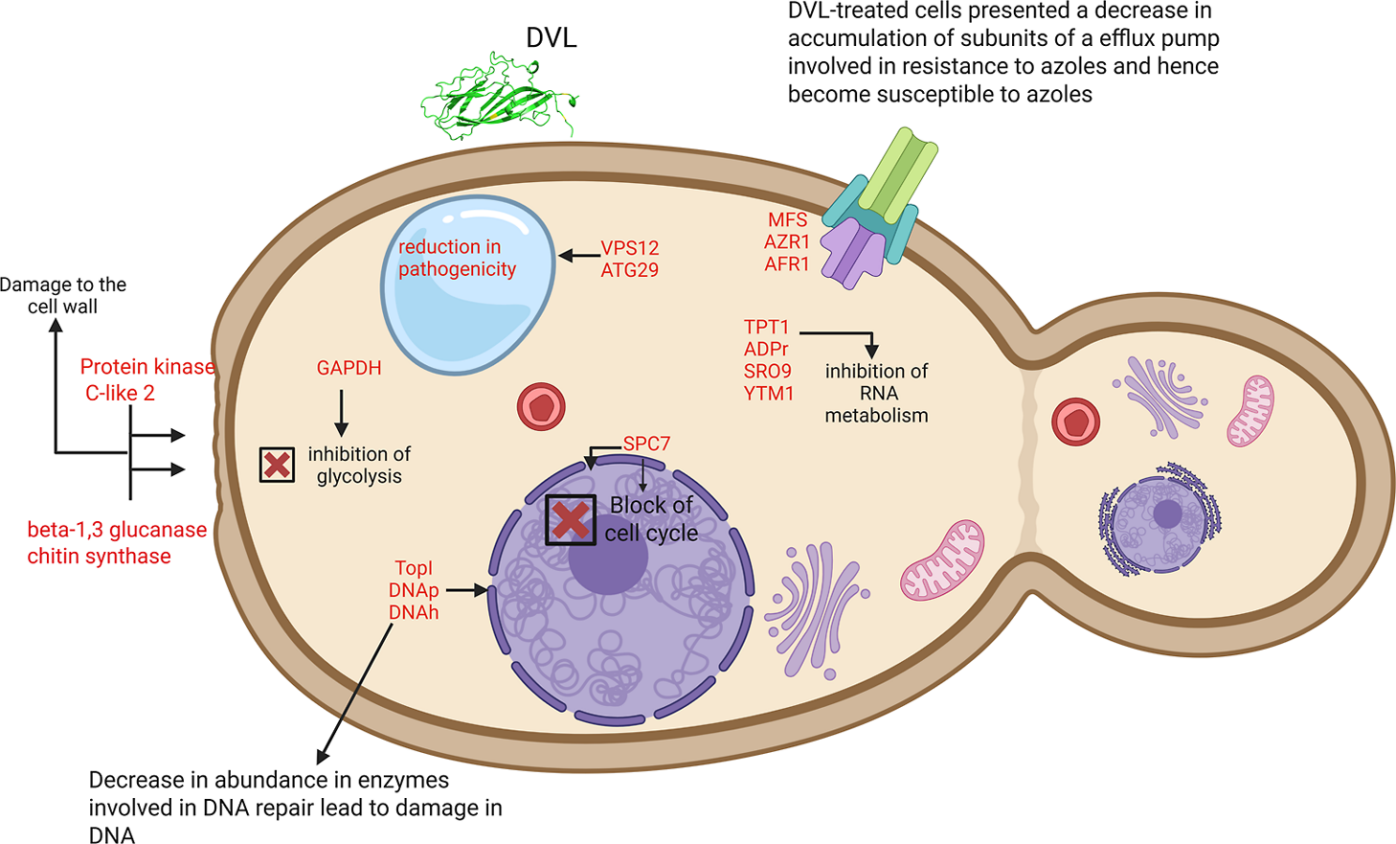
–Overview of the effect caused by DVL on protein profile of Candida cells. Proteomic analysis revealed that DVL induced several changes in cellular pathways critical to cell survival.

The fungal cell wall is a validated target for antifungal drug development. Inhibiting 1,3-β-glucan synthase, a key component of cell wall synthesis, is a particular focus, as demonstrated by the success of lipopeptide-based antifungals like echinocandins and pneumocandins.^31,32^

Among the proteins involved in cell wall synthesis, chitin synthase was exclusively identified in DVL-treated cells (Table S2). As a key enzyme in chitin biosynthesis and a known antifungal target, its up-accumulation corroborates the damage caused by DVL to the cell wall, and that cell is trying to recover from such damage.^33,34^ Chitin synthesis likely represents an adaptive response to reinforce the cell wall in response to DVL treatment, corroborating our previous hypothesis (Figure 4).

Cell wall mannoprotein was also identified as a unique protein in DVL-treated cells. Yeast cell wall mannoproteins play crucial roles in cell wall organization, size, and shape, and we know that disruption of these mannoproteins can cause alterations in cell morphology.^35^ In addition, specifically in *C. albicans*, they are associated with adhesion, drug resistance, and virulence.^36^ It is important to notice that DVL is a glucose/mannose-binding lectin. Therefore, it is feasible to suggest that DVL interacts with mannoproteins, inhibiting them; nonetheless, the increase in mannoproteins could be a response of *C. albicans* to the blockage of mannoproteins by DVL.

Protein kinase C-like 2 was exclusively detected in control cells (Table S1). Protein kinases play crucial roles in fungal pathogenicity by acting on cellular processes, including cell wall homeostasis, morphogenesis, and responses to the cell wall and membrane stress (Figure 4).^37^ The absence of this protein in the treated cells suggests that DVL interferes with normal signaling pathways, possibly causing a suppression of the activity or expression of the protein kinase, which ends up hindering the basic functions of this protein, such as repairing damage to the cell wall.

Given that DVL is a carbohydrate-binding lectin, its interaction with intracellular targets suggests the involvement of an internalization mechanism. Plant lectins like concanavalin A and ricin are internalized and transported to various subcellular locations, including the endoplasmic reticulum and nucleus.^37,38^ Helja, the lectin from *Helianthus annus*, was able to accumulate inside the cytoplasm of spores from *Sclerotinia sclerotiorum* by interaction involving the carbohydrate-binding site. In the presence of mannose, Helja lost the ability to reach the cytoplasm.^39^ While the internalization of DVL has not been directly observed in this study, these parallels suggest that DVL may exploit similar pathways to exert intracellular effects. Further studies using fluorescence labeling or endocytosis inhibitors could clarify the mechanism of DVL uptake.

### 3.3 Cell Cycle Proteins

The cell cycle plays an essential role in the growth and reproduction of pathogenic fungi, directly influencing their life cycles. Therefore, cell cycle components, such as proteins, have significant potential as therapeutic targets for developing antifungal strategies to combat these infections.^38,39^ In this group of proteins, we find the Kinetochore protein Spc7 present in both groups, but with a reduction in abundance in DVL-treated cells. Kinetochore protein Spc7 is a kinetochore complex component essential for accurate chromosome segregation during cell division.

Kinetochore protein Spc7 is a component of the kinetochore, a large cluster of proteins that plays an important role in chromosome segregation and cell cycle progression, regulating microtubule binding and the spindle checkpoint.^40,41^ Another important point is that the Spc7 protein is crucial for maintaining the integrity of the mitotic spindle and is responsible for binding kinetochore complexes in yeast cell division (Figure 4). The lack of this protein results in significant defects in the spindle structure.^42^ Negative regulation of this protein can affect cell division and, consequently, the viability of these cells, acting on reproduction and virulence processes, which is proven when observing the antifungal activity of DVL.

### 3.4 Transmembrane Transport Proteins

In this group of proteins, some stand out individually, both in control and treated cells. In control cells, the Intermembrane lipid transfer protein VPS13, whose function is linked to lipid transport between organelles, is essential for maintaining and functioning cellular compartments such as vacuoles.^43^ The absence of this protein in DVL-treated cells suggests impairments in transport and intracellular organization, in addition to impacting the maintenance of vacuoles that perform various cellular functions, some crucial for pathogenicity, and the interruption of these functions may be indicative of studies for antifungal agents.^44^

The Autophagy-related protein 29 (ATG29) was identified only in control cells. We understand that autophagy, in eukaryotes, is a regenerative process in which cells can recover and utilize damaged organelles and proteins.^45^ The absence of ATG29 in DVL-treated cells indicates an interference or even inhibition of the autophagic process, interfering with the ability of cells to respond to stress and maintain cellular balance. Moreover, according to Liu et al.,^46^ inhibition of autophagy in *C. albicans* leads to decreased biofilm formation and antifungal resistance.

Another protein is the MFS-type transporter, unique from DVL-treated cells within this category. MFS proteins are considered the largest superfamily of secondary active transporters, catalyzing the transport of a wide range of substrates in both directions across the membrane.^47,48^ Within fungal activity, MFS proteins in *C. albicans* play a significant role in multidrug resistance, including azoles.^49^ In our findings, this response is complemented by the expression of the Azole resistance protein 1 (AZR1) and Cycloheximide resistance protein, also in cells treated with DVL. The simultaneous expression of these three proteins may suggest reinforcing this resistance mechanism, where the cell responds to this stress situation.

AZR1 is a transporter protein involved in resistance to azoles and low-chain organic acids. In the presence of azoles, it encodes the action of efflux pumps, which are a significant resistance mechanism for *C. albicans*.^50,51^ This drug acts by blocking the biosynthesis of ergosterol, the sterol component of the fungal plasma membrane.^52^ Correlating with our previous study,^9^ DVL treatment resulted in a 58% reduction in ergosterol biosynthesis. The expression of the AZR1 protein is suggestive of a possible adaptive response of the attacked cell, in which it tries to compensate or neutralize the disturbance caused by ergosterol synthesis, potentially trying to reduce the effectiveness of the treatment.

The ABC multidrug transporter AFR1, a multidrug transporter protein, was exclusively detected in the control cells (Table S1). The AFR1 is largely involved in *Cryptococcus neoformans* resistance to azoles (e.g., fluconazole) (Figure 4).^53^ It makes total sense that the presence of AFR1 is only in control cells (Table S1). *Candida* cells use transporter proteins (e.g., ABC) to resist cell death by antifungal drugs and facilitate cell survival.^54^ The absence of AFR1 in DVL-treated *C. albicans* cells suggests that a previous azole-resistant strain may have regressed to susceptible status (Figure 4). The azole groups display their mechanism of action within the cytoplasm by inhibiting the cytochrome P450 lanosterol 14α-demethylase and, hence, the ergosterol biosynthesis.^55^ In turn, the resistance mechanism to azoles is based on reducing intracellular entrance and concentration by efflux pumps,^55^ such as AFR1. The absence of AFR1 in DVL-treated cells suggests susceptibility to azole drugs and synergism between DVL and azole drugs (Figure 4).

### 3.5 DNA Repair Proteins

DNA repair proteins recognize damage through interaction with damaged DNA and undergo conformational changes, resolving the speed-stability paradox through atypical diffusion.^56^ Such proteins, including Topoisomerase I (TopI), DNA polymerase (DNAp), and DNA helicase (DNAh), were exclusively found in control *C. albicans* cells and absent in DVL-treated cells (Figure 4).

Topoisomerases are proteins that alter the topological structure of DNA, which are essential for replication, transcription, and cell repair, and are commonly found in *C. albicans* cells in type 1 and type 2 forms. Their inhibition is the target of many antifungal agents.^57,58^ Topoisomerase I (TopI) is induced during cellular stress, preventing DNA damage and cell death by apoptosis.^59^ The absence of this protein in DVL-treated cells suggests an interruption in the DNA-protective mechanisms, leading to DNA damage and cell death. For example, topoisomerases have an important role in the protection of DNA from oxidative damage induced by ROS.^60,61^

Based on that, we hypothesized that *C. albicans* treated with DVL would suffer DNA damage. In a previous study, we showed that DVL induced high accumulation in *C. albicans* cells. Along with the absence of topoisomerase, high ROS accumulation may damage DNA in *C. albicans* cells.

DNA polymerase and DNA helicase are also essential proteins in DNA replication, repair, and recombination, and play an important role in genome maintenance and pathogenicity in *C. albicans*. The absence of these two proteins in DVL-treated cells directly impacts cell viability since both work together to ensure the fidelity of the DNA replication process. With their absence, errors may occur in the replication processes, contributing to genomic instability and cell death.^62–64^ These findings reinforce the anti-*candida* potential of DVL since it can interfere with the inhibition of proteins essential for the proper functioning of DNA repair and replication (Figure 4).

### 3.6 Proteins Related to Metabolism and Energy

In this group, proteomic analysis revealed the presence of the protein Glyceraldehyde-3-phosphate dehydrogenase (GAPDH) exclusively in DVL-treated cells (Table S2, Figure 4). GAPDH is well-known for its central role in energy metabolism and the production of ATP through glycolysis.^65^ When associated with the cell wall in *C. albicans*, it plays a critical role in pathogen-host interaction, mediating the adhesion of the fungus to components of the extracellular matrix, such as fibronectin and laminin, facilitating the attachment and spread of the fungal infection, and this attachment capacity is amplified under stress conditions.^66–68^

The exclusive presence of GAPDH in treated cells may indicate a response to metabolic stress, in which maintaining energy homeostasis is vital for cell development and resistance to stressful environments.^69^ This adaptive response supports energy maintenance and may also be related to the pathogenic functions of adhesion and invasion, indicating a multifunctional role of GAPDH under the influence of DVL.

### 3.7 Proteins Related to RNA Regulation and Processing Factors

RNA processing is essential for fungal cell viability, as it regulates protein synthesis and consequently plays a critical role in fungal pathogenesis.^70,71^ In this group, we identified the protein Putative tRNA 2′-phosphotransferase (Tpt1) exclusively in DVL-treated cells (Table S2). The Tpt1 is essential for tRNA splicing and maturation in fungi, can mediate phospho-ADP ribosylation (ADPr), plays a key role in maintaining protein efficiency under adverse conditions, and, due to this characteristic, is considered a potential antifungal target (Figure 4).^72^ The exclusive presence of this protein in DVL-treated cells reflects a cell survival strategy, enhancing the fungus’s ability to resist the stressful effects of the lectin.

In the group of control proteins (Table S1), we found two interesting proteins: the RNA-binding protein SRO9 and the Ribosome biogenesis protein YTM1 (Figure 4). SRO9 is a protein that plays a role in bud outgrowth and the organization of actin filaments, contributing to polarized growth in yeast.^73,74^ The absence of this protein in DVL-treated cells suggests a disruption in the normal processes of polarization and cell division, potentially as part of a defensive strategy or stress response.

YTM1 is a protein involved in ribosome biogenesis and is crucial for the assembly and maturation of pre-60S ribosomes, playing a key role in cell proliferation.^75^ The absence of this protein in DVL-treated cells suggests an adaptive response where treatment with the lectin may influence the modification of ribosome biogenesis and indicate a possible reduction in protein translation capacity to conserve resources under stressful conditions. It has been well reported that inhibiting biogenesis in yeast leads to a delay in cell proliferation and a decrease in translation capacity.^76^

### 3.8 Proteins Related to Intracellular Protein Transport

Dynamin-like GTPase MGM1 is a protein exclusive to cells treated with DVL. This protein is crucial for maintaining mitochondrial morphology and function, cell cycle progression, hyphae development, and virulence in *C. albicans*.^77^ The expression of this protein in cells treated with DVL may be a direct response to the effects induced by this lectin. As reported previously, DVL induces the overexpression of reactive oxygen species (ROS) and disrupts energy metabolism,^9^ and the function of MGM1 in maintaining mitochondrial morphology and function is crucial (Figure 4). Mitochondria are important for redox balance and energy production, as well as ROS detoxification.^78^ In response to the oxidative stress and metabolic disruption caused by DVL, the expression of MGM1 may be an attempt to compensate for the damage and preserve cell viability under lectin-induced stress conditions.

### 3.9 Defense and Stress-Related Proteins

In this group, it was identified the Catalase domain-containing protein exclusively in the cells treated with DVL (Table S2). Catalase is an antioxidant protein in fungi, preventing oxidative stress.^79^ The exclusive presence of this protein in the DVL-treated cells suggests an attempt to respond to the oxidative stress that we already know the lectin causes in these cells. However, as we saw in our previous study, there is a decrease in catalase and peroxidase activities, which would normally break down accumulated hydrogen peroxide, in *C. albicans* cells treated with DVL.^9^ The high accumulation of catalase in DVL-treated cells is a response to the high accumulation of ROS revealed in our previous work.^9^

Exclusively in the control cells (Table S1), we identified the Ceramide-binding protein SVF1. Ceramides are essential for various cellular functions and can affect membrane structure and function as signaling molecules that promote cell death; SVF1, by binding to ceramides, probably helps regulate how cells respond to stress.^80^ The absence of SVF1 in cells exposed to DVL suggests a possible vulnerability of these cells to stress and deregulation of ceramide-mediated signals, which may facilitate pathological processes such as cell death.

### 3.10 Carbohydrate Metabolism Proteins

Exclusively in the control cells (Table S1), we identified the protein β-*N*-acetylhexosaminidase. This protein plays a crucial role in glycoprotein metabolism, being functional for cell wall renewal and chitin synthesis, as well as acting on the structural integrity of the fungus; it also acts in cell differentiation throughout its growth cycle.^81,82^ Its absence in the treated cells suggests a possible adaptive response to the stress induced by DVL, which, as a consequence, can weaken the cell wall, making it a therapeutic target.

In DVL-treated cells (Table S2), we found the protein Mannan endo-1,6-α-mannosidase. This protein can degrade mannans, mannose-rich polysaccharides present in the yeast cell wall, and this degradation results in free mannose and resistant polymers.^83,84^ The expression of this protein in DVL-treated cells suggests that it is involved in cell wall remodeling mechanisms in the face of deformations caused by DVL attack.^9,85^ As DVL has an affinity for mannose, we suggest that the release of free mannose may act by saturating the lectin binding sites, potentially preventing the lectin from binding to the cell wall.

## 4 Conclusions

This study demonstrates that the lectin DVL significantly affects the protein profile of *C. albicans,* modulating key proteins involved in important cellular pathways related to cell wall synthesis, oxidative stress response, energy metabolism, and DNA repair. While proteomic analysis provides a broad overview of these changes, further functional studies are needed to confirm the mechanisms involved. Despite these limitations, our findings highlight the potential of DVL as a promising alternative to conventional antifungal therapies.

## Supplementary Material



## Notes

The Supporting Information reports the detailed information on all proteins identified by proteomic analysis (PDF)
