# Supplementary material for: Proteomic Analysis Revealed that DVL, a Lectin Purified from Dioclea violacea Seeds, Induced a Change in the Protein Profile of Candida albicans
Source: ACS Omega. 2025 Aug 29;10(35):40046–55. doi: 10.1021/acsomega.5c04868 (PMC12423798; doi:10.1021/acsomega.5c04868)
Supplement: Supplementary file 1 [file ao5c04868_si_001.pdf]

SUPPLEMENTARY MATERIAL

Proteomic analysis revealed that DVL, a lectin purified from *Dioclea violacea* seeds, induced a change in the protein profile of *Candida albicans*

Romério R. S. Silva[1]; Maria H. C. Santos[1]; Ana L. E. Santos[2]; Cleverson D. T. Freitas[1]; Rômulo F. Carneiro[3]; Celso S. Nagano[3]; Felipe P. Mesquita[4,5], Pedro F. N. Souza[4,5,6,7*]; Claudener S. Teixeira[2*].

[1] Department of Biochemistry and Molecular Biology, Federal University of Ceará, Fortaleza 60451-970, CE, Brazil.
[2] Medical School, Federal University of Cariri, Barbalha, Ceará, Brazil.
[3] Department of Fisheries Engineering, Federal University of Ceará (UFC), Fortaleza, CE, Brazil
[4] Laboratory of Pharmacogenetics, Center for Drug Research and Development (NPDM), Department of Physiology and Pharmacology, Federal University of Ceará, Fortaleza 60430-275, CE, Brazil.
[5] Laboratory of Bioinformatics Applied to Human Health, Center for Drug Research and Development (NPDM), Federal University of Ceará, Fortaleza, Brazil.
[6] National Institute of Science and Technology in Human Pathogenic Fungi (FunVir), Brazil.
[7] Visiting Researcher at the Cearense Foundation to Support Scientific and Technological Development.
[5] Center for Agricultural Sciences and Biodiversity, Federal University of Cariri, Crato 63130-025, Brazil.

***Corresponding authors**:

Pedro F. N. Souza (pedrofilhobio@gmail.com) at Laboratory of Bioinformatics Applied to Human Health Drug Research at the Development Center, Department of Physiology and Pharmacology, Federal University of Ceará, Fortaleza, Ceará, Brazil.

Claudener S. Teixeira (claudener@gmail.com) at Center for Agricultural Sciences and Biodiversity, Federal University of Cariri, Crato 63130-025, Brazil

Supplementary Table S1 - Unique proteins identified in the control group by ESI-LC-MS/MS

Protein Name	ID UniProt	Organism Reference	Cellular Compartment
**Sporulation**			
Sporulation-specific protein 71	Q03868	*Saccharomyces cerevisiae* (strain ATCC 204508 / S288c)	Prospore membrane
**Transcription regulation**			
Transcriptional coactivator HFI1/ADA1	Q12060	*Saccharomyces cerevisiae* (strain ATCC 204508 / S288c)	Nucleus
Putative transcriptional activator MSA2	P36157	*Saccharomyces cerevisiae* (strain ATCC 204508 / S288c)	Nucleus
Protein HIR1	Q74ZN0	*Ashbya gossypii* (strain ATCC 10895 / CBS 109.51 / FGSC 9923 / NRRL Y-1056)	Nucleus
Serine/threonine-protein kinase SSN3	Q5AHK2	*Candida albicans* (strain SC5314 / ATCC MYA-2876)	CKM complex
Transcription factor prr1	O14283	*Schizosaccharomyces pombe* (strain 972 / ATCC 24843)	Nucleus
DNA-directed RNA polymerase III subunit rpc-3	Q8WZW7	*Neurospora crassa* (strain ATCC 24698 / 74-OR23-1A / CBS 708.71 / DSM 1257 / FGSC 987)	Nucleus
Actin-like protein ARP6	Q74ZV8	*Ashbya gossypii* (strain ATCC 10895 / CBS 109.51 / FGSC 9923 / NRRL Y-1056)	Cytoplasm
**Intracellular Protein Transport**			
Protein transport protein SEC31	P38968	*Saccharomyces cerevisiae* (strain ATCC 204508 / S288c)	Endoplasmic reticulum
ENTH domain-containing protein	A0A2H3GR05	*Fusarium oxysporum f. sp. radicis-cucumerinum*	Membrane
Importin N-terminal domain-containing protein	A0A2H3H1T0	*Fusarium oxysporum f. sp. radicis-cucumerinum*	Nucleus
Vacuolar protein sorting/targeting protein 10	Q2HAB1	*Chaetomium globosum* (strain ATCC 6205 / CBS 148.51 / DSM 1962 / NBRC 6347 / NRRL 1970)	Membrane

ER-retained PMA1-suppressing protein 1	P40557	*Saccharomyces cerevisiae* (strain ATCC 204508 / S288c)	Endoplasmic reticulum
Autophagy-related protein 11	Q7S055	*Neurospora crassa* (strain ATCC 24698 / 74-OR23-1A / CBS 708.71 / DSM 1257 / FGSC 987)	Membrane
Coatomer subunit delta	P43621	*Saccharomyces cerevisiae* (strain ATCC 204508 / S288c)	Golgi membrane
**Gene regulation**			
Protein SPT3	P06844	*Saccharomyces cerevisiae* (strain ATCC 204508 / S288c)	Nucleus
Histone deacetylase RPD3	P32561	*Saccharomyces cerevisiae* (strain ATCC 204508 / S288c)	Nucleus
La protein homolog	P33399	*Saccharomyces cerevisiae* (strain ATCC 204508 / S288c)	Nucleus
Bromodomain-containing factor 2	Q07442	*Saccharomyces cerevisiae* (strain ATCC 204508 / S288c)	Nucleus
Exosome complex component SKI6	P46948	*Saccharomyces cerevisiae* (strain ATCC 204508 / S288c)	Nucleus
Transcriptional regulatory protein SIN3	P22579	*Saccharomyces cerevisiae* (strain ATCC 204508 / S288c)	Nucleus
E3 ubiquitin-protein ligase HEL2	Q05580	*Saccharomyces cerevisiae* (strain ATCC 204508 / S288c)	Cytoplasm
Zn(2)-C6 fungal-type domain-containing protein	A0A2H3HHA4	*Fusarium oxysporum f. sp. radicis-cucumerinum*	Nucleus
C3H1-type domain-containing protein	A0A2H3GWC8	*Fusarium oxysporum f. sp. radicis-cucumerinum*	Nucleus
F-box domain-containing protein	A0A2H3GUI5	*Fusarium oxysporum f. sp. radicis-cucumerinum*	Cytoplasm
Putative COX1/OXI3 intron 2 protein	P03876	*Saccharomyces cerevisiae* (strain ATCC 204508 / S288c)	Membrane
Silencing boundary-establishment protein FUB1-like protein	Q9UTI1	*Schizosaccharomyces pombe* (strain 972 / ATCC 24843)	Nucleus
**Cellular signaling**			
DOCK-like protein 1	Q06409	*Saccharomyces cerevisiae* (strain ATCC 204508 / S288c)	Cytoplasm

GTPase-activating protein BEM2/IPL2	P39960	*Saccharomyces cerevisiae* (strain ATCC 204508 / S288c)	Cytoplasm
**DNA repair**			
Holliday junction resolvase YEN1	P40028	*Saccharomyces cerevisiae* (strain ATCC 204508 / S288c)	Nucleus
Protein MLP2	P40457	*Saccharomyces cerevisiae* (strain ATCC 204508 / S288c)	Nucleus
Telomere length regulation protein ELG1	Q12050	*Saccharomyces cerevisiae* (strain ATCC 204508 / S288c)	Nucleus
Protein HIM1	Q06674	*Saccharomyces cerevisiae* (strain ATCC 204508 / S288c)	Nucleus
DNA-dependent ATPase MGS1	P40151	*Saccharomyces cerevisiae* (strain ATCC 204508 / S288c)	Nucleus
Structural maintenance of chromosomes protein 5	A0A2H3H0P9	*Fusarium oxysporum f. sp. radicis-cucumerinum*	Nucleus
DNA helicase	A0A2H3G9Q3	*Fusarium oxysporum f. sp. radicis-cucumerinum*	Nucleus
Structure-specific endonuclease subunit SLX4	Q2HCM6	*Chaetomium globosum* (strain ATCC 6205 / CBS 148.51 / DSM 1962 / NBRC 6347 / NRRL 1970)	Nucleus
DNA polymerase epsilon catalytic subunit A	Q6CUS7	*Kluyveromyces lactis* (strain ATCC 8585 / CBS 2359 / DSM 70799 / NBRC 1267 / NRRL Y-1140 / WM37)	Nucleus
DNA polymerase	P05468	*Kluyveromyces lactis* (strain ATCC 8585 / CBS 2359 / DSM 70799 / NBRC 1267 / NRRL Y-1140 / WM37)	Nucleus
ATP-dependent helicase SGS1	P35187	*Saccharomyces cerevisiae* (strain ATCC 204508 / S288c)	Nucleus
Restriction of telomere capping protein 1	Q6CME1	*Kluyveromyces lactis* (strain ATCC 8585 / CBS 2359 / DSM 70799 / NBRC 1267 / NRRL Y-1140 / WM37)	Vacuole
Topoisomerase 1-associated factor 1	A5DJ85	*Meyerozyma guilliermondii* (strain ATCC 6260 / CBS 566 / DSM 6381 / JCM 1539 / NBRC 10279 / NRRL Y-324)	Nucleus
**Metabolism and energy**			
Phosphatidylglycerophosphatase GEP4, mitochondrial	P38812	*Saccharomyces cerevisiae* (strain ATCC 204508 / S288c)	Mitochondrial matrix
Acetyl-CoA carboxylase	Q00955	*Saccharomyces cerevisiae* (strain ATCC 204508 / S288c)	Cytoplasm

Cytochrome c oxidase assembly protein COX15	P40086	*Saccharomyces cerevisiae* (strain ATCC 204508 / S288c)	Mitochondria
**Protein biosynthesis**			
Tryptophan--tRNA ligase, cytoplasmic	Q12109	*Saccharomyces cerevisiae* (strain ATCC 204508 / S288c)	Cytoplasm
WD repeat-containing protein JIP5	Q06214	*Saccharomyces cerevisiae* (strain ATCC 204508 / S288c)	Nucleus
Aspartate--tRNA ligase, cytoplasmic	P04802	*Saccharomyces cerevisiae* (strain ATCC 204508 / S288c)	Cytoplasm
Isoleucine--tRNA ligase, cytoplasmic	P09436	*Saccharomyces cerevisiae* (strain ATCC 204508 / S288c)	Cytoplasm
Ribosome biogenesis protein RLP7	P40693	*Saccharomyces cerevisiae* (strain ATCC 204508 / S288c)	Nucleolus
54S ribosomal protein L10, mitochondrial	P36520	*Saccharomyces cerevisiae* (strain ATCC 204508 / S288c)	Mitochondria
Phenylalanine--tRNA ligase alpha subunit	P15625	*Saccharomyces cerevisiae* (strain ATCC 204508 / S288c)	Cytoplasm
Elongation factor 1-alpha	A0A2C8 CZL3	*Saccharomyces cerevisiae* (strain ATCC 204508 / S288c)	Cytoplasm
**Transmembrane transporters**			
Metal resistance protein YCF1	P39109	*Saccharomyces cerevisiae* (strain ATCC 204508 / S288c)	Membrane
Membrane-anchored lipid-binding protein LAM4	P38800	*Saccharomyces cerevisiae* (strain ATCC 204508 / S288c)	Plasm membrane
Protein transport protein SBH1	P52870	*Saccharomyces cerevisiae* (strain ATCC 204508 / S288c)	Membrane
Fluconazole resistance protein	P38124	*Saccharomyces cerevisiae* (strain ATCC 204508 / S288c)	Plasm membrane
MFS domain-containing protein	A0A2H3 HZD2	*Fusarium oxysporum* f. sp. *radicis-cucumerinum*	Membrane
Autophagy-related protein 29	A0A2H3 HZZ7	*Fusarium oxysporum* f. sp. *radicis-cucumerinum*	Phagophore

DUF5672 domain-containing protein	A0A2H3GCI3	*Fusarium oxysporum* f. sp. *radicis-cucumerinum*	Membrane
Major facilitator copper-regulated transporter crmC	Q4WYN4	*Aspergillus fumigatus* (strain ATCC MYA-4609 / CBS 101355 / FGSC A1100 / Af293)	Plasm membrane
Mitochondrial thiamine pyrophosphate carrier 1	A3LVX1	*Scheffersomyces stipitis* (strain ATCC 58785 / CBS 6054 / NBRC 10063 / NRRL Y-11545)	Mitochondrial membrane
ABC multidrug transporter AFR1	P9WEU4	*Cryptococcus gattii serotype* B (strain R265)	Plasm membrane
Intermembrane lipid transfer protein VPS13	Q07878	*Saccharomyces cerevisiae* (strain ATCC 204508 / S288c)	Endosome
**Cell cycle**			
PHO85 cyclin-1	P24867	*Saccharomyces cerevisiae* (strain ATCC 204508 / S288c)	Cytoplasm
Negative regulator of mitosis	P24686	*Emericella nidulans* (strain FGSC A4 / ATCC 38163 / CBS 112.46 / NRRL 194 / M139)	Cytoplasm
Protein STU1	Q75B70	*Ashbya gossypii* (strain ATCC 10895 / CBS 109.51 / FGSC 9923 / NRRL Y-1056)	Nucleus
**Intracellular regulation**			
Vacuolar protein sorting-associated protein 13	Q07878	*Saccharomyces cerevisiae* (strain ATCC 204508 / S288c)	Endosome
Protein SBE22	P38814	*Saccharomyces cerevisiae* (strain ATCC 204508 / S288c)	Cytoplasm
Protein kinase domain-containing protein	A0A2H3HHU3	*Fusarium oxysporum* f. sp. *radicis-cucumerinum*	Cytoplasm
Pep3_Vps18 domain-containing protein	A0A2H3H0M4	*Fusarium oxysporum* f. sp. *radicis-cucumerinum*	Membrane
Nuclear envelope protein YPR174C	Q06616	*Saccharomyces cerevisiae* (strain ATCC 204508 / S288c)	Cytoplasm
Mitochondrial distribution and morphology protein 10	C1GVA2	*Paracoccidioides lutzii* (strain ATCC MYA-826 / Pb01)	Mitochondria
**Metabolism and synthesis of amino acids**			

Glutamine synthetase	P32288	*Saccharomyces cerevisiae* (strain ATCC 204508 / S288c)	Cytoplasm
Dipeptidase	A0A2H3 GEE7	*Fusarium oxysporum* f. sp. *radicis-cucumerinum*	Cytoplasm
Peroxisomal membrane protein PEX32	P38292	*Saccharomyces cerevisiae* (strain ATCC 204508 / S288c)	Peroxisomal membrane
Iterative polyketide synthase CazM	A0A0K0 MCJ4	*Chaetomium globosum* (strain ATCC 6205 / CBS 148.51 / DSM 1962 / NBRC 6347 / NRRL 1970)	Unknown
Short chain dehydrogenase CPUR_05429	M1W270	*Claviceps purpurea* (strain 20.1)	Unknown
FAD-dependent monooxygenase atnK	A0A455L LW7	*Arthrinium* sp.	Membrane
Arginine biosynthesis bifunctional protein ArgJ, mitochondrial	B8PH83	*Postia placenta* (strain ATCC 44394 / Madison 698-R)	Mitochondrial matrix
Pentafunctional AROM polypeptide	Q0V3H0	*Phaeosphaeria nodorum* (strain SN15 / ATCC MYA-4574 / FGSC 10173)	Cytoplasm
Probable cytosolic iron-sulfur protein assembly protein 1	Q5BDJ5	*Emericella nidulans* (strain FGSC A4 / ATCC 38163 / CBS 112.46 / NRRL 194 / M139)	Cytoplasm
3-isopropylmalate dehydratase	Q74ZM9	*Ashbya gossypii* (strain ATCC 10895 / CBS 109.51 / FGSC 9923 / NRRL Y-1056)	Cytoplasm
Probable Xaa-Pro aminopeptidase PEPP	E4ZHV7	*Leptosphaeria maculans* (strain JN3 / isolate v23.1.3 / race Av1-4-5-6-7-8)	Cytoplasm
**Transferase**			
Putative protein lysine methyltransferase SET5	P38890	*Saccharomyces cerevisiae* (strain ATCC 204508 / S288c)	Cytoplasm
Choline kinase	P20485	*Saccharomyces cerevisiae* (strain ATCC 204508 / S288c)	Cytoplasm
Protein arginine methyltransferase NDUFAF7	A0A2H3 GT25	*Fusarium oxysporum f. sp. radicis-cucumerinum*	Mitochondria
N-acetyltransferase domain-containing protein	A0A2H3 H414	*Fusarium oxysporum f. sp. radicis-cucumerinum*	Unknown

Probable E3 ubiquitin-protein ligase TOM1	Q756G2	*Ashbya gossypii* (strain ATCC 10895 / CBS 109.51 / FGSC 9923 / NRRL Y-1056)	Cytoplasm
Polyketide synthase 1	A0A2U8 NET4	*Metarhizium album* (strain ARSEF 1941)	Cytoplasm
Methyltransferase tpcH	Q4WQZ0	*Aspergillus fumigatus* (strain ATCC MYA-4609 / CBS 101355 / FGSC A1100 / Af293)	Cytoplasm
D-lysergyl-peptide-synthetase subunit 3	M1VVW 6	*Claviceps purpurea* (strain 20.1)	Cytoplasm
AdoMet-dependent rRNA methyltransferase SPB1	Q6C9Q1	*Yarrowia lipolytica* (strain CLIB 122 / E 150)	Nucleolus
GPI mannosyltransferase 4	Q5BAX7	*Emericella nidulans* (strain FGSC A4 / ATCC 38163 / CBS 112.46 / NRRL 194 / M139)	Cytoplasm
Tryptophan dimethylallyltransferase nptA	C8VEJ5	*Emericella nidulans* (strain FGSC A4 / ATCC 38163 / CBS 112.46 / NRRL 194 / M139)	Cytoplasm
Hexaprenyl pyrophosphate synthase, mitochondrial	P18900	*Saccharomyces cerevisiae* (strain ATCC 204508 / S288c)	Mitochondria
5-aminolevulinate synthase, mitochondrial	P38092	*Emericella nidulan*s (strain FGSC A4 / ATCC 38163 / CBS 112.46 / NRRL 194 / M139)	Mitochondria
**Regulation Factor and RNA Processing**			
RNA-binding protein SRO9	P25567	*Saccharomyces cerevisiae* (strain ATCC 204508 / S288c)	Nucleus
MI domain-containing protein	A0A2H3 H7M0	*Fusarium oxysporum f. sp. radicis-cucumerinum*	Nucleus
ATP-dependent RNA helicase mss116, mitochondrial	O13622	*Schizosaccharomyces pombe* (strain 972 / ATCC 24843)	Mitochondria
ATP-dependent RNA helicase MAK5	Q757I6	*Ashbya gossypii* (strain ATCC 10895 / CBS 109.51 / FGSC 9923 / NRRL Y-1056)	Nucleolus
Ribosome biogenesis protein YTM1	A6R3K5	*Ajellomyces capsulatus* (strain NAm1 / WU24)	Nucleolus

ATP-dependent RNA helicase rok1	Q2UQW3	*Aspergillus oryzae* (strain ATCC 42149 / RIB 40)	Nucleolus
ATP-dependent RNA helicase DBP3	Q2GUI4	*Chaetomium globosum* (strain ATCC 6205 / CBS 148.51 / DSM 1962 / NBRC 6347 / NRRL 1970)	Nucleolus
**Oxireductase**			
Amino_oxidase domain-containing protein	A0A2H3HA32	*Fusarium oxysporum f. sp. radicis-cucumerinum*	Unknown
FAD-binding FR-type domain-containing protein	A0A2H3GTL6	*Fusarium oxysporum f. sp. radicis-cucumerinum*	Membrane
3-hydroxyanthranilate 3,4-dioxygenase 1	Q2UHT9	*Aspergillus oryzae* (strain ATCC 42149 / RIB 40)	Cytoplasm
Cytochrome P450 monooxygenase 75	F1SY74	*Postia placenta* (strain ATCC 44394 / Madison 698-R)	Membrane
Protein YIM1	A7TH23	*Vanderwaltozyma polyspora* (strain ATCC 22028 / DSM 70294 / BCRC 21397 / CBS 2163 / NBRC 10782 / NRRL Y-8283 / UCD 57-17)	Mitochondria
Highly reducing polyketide synthase VdtX	A0A443HK66	*Byssochlamys spectabilis*	Unknown
**Carbohydrate metabolism**			
Beta-N-acetylhexosaminidase	A0A2H3GVW6	*Fusarium oxysporum f. sp. radicis-cucumerinum*	Lysosomes
Galactan endo-beta-1,3-galactanase	F7J1C8	*Flammulina velutipes*	Cytoplasm
Putative dipeptidase NECHADRAFT_87110	C7ZIE1	*Fusarium vanettenii* (strain ATCC MYA-4622 / CBS 123669 / FGSC 9596 / NRRL 45880 / 77-13-4)	Membrane
**Lipid biosynthesis**			
Very-long-chain 3-oxoacyl-CoA reductase	A0A2H3GJ31	*Fusarium oxysporum f. sp. radicis-cucumerinum*	Endoplasmic reticulum.
Fatty acid synthase subunit alpha	Q765N2	*Lachancea kluyveri*	Sintase complex

Tafazzin	Q06510	*Saccharomyces cerevisiae* (strain ATCC 204508 / S288c)	Mitochondrial membrane
Altered inheritance of mitochondria protein 6	C5DD45	*Lachancea thermotolerans* (strain ATCC 56472 / CBS 6340 / NRRL Y-8284)	Unknown
Peroxisomal hydratase-dehydrogenase-epimerase	Q01373	*Neurospora crassa* (strain ATCC 24698 / 74-OR23-1A / CBS 708.71 / DSM 1257 / FGSC 987)	Peroxisome
**Nucleotide metabolism**			
Metallophos domain-containing protein	A0A2H3G086	*Fusarium oxysporum f. sp. radicis-cucumerinum*	Nucleus
AB hydrolase-1 domain-containing protein	A0A2H3GRS7	*Fusarium oxysporum f. sp. radicis-cucumerinum*	Nucleus
Kynureninase 2	A1CHT0	*Aspergillus clavatus* (strain ATCC 1007 / CBS 513.65 / DSM 816 / NCTC 3887 / NRRL 1 / QM 1276 / 107)	Cytoplasm
**Cell Redox Homeostasis**			
FAD-binding PCMH-type domain-containing protein	A0A2H3HIF0	*Fusarium oxysporum f. sp. radicis-cucumerinum*	Cytoplasm
**Stress and Defense Response**			
Cytochrome P450 monooxygenase stcB	Q12608	*Emericella nidulans* (strain FGSC A4 / ATCC 38163 / CBS 112.46 / NRRL 194 / M139)	Membrane
Hybrid PKS-NRPS synthetase tasS	A0A0F7GFS4	*Hapsidospora irregularis*	Cytoplasm
Probable oxidoreductase yanE	G3Y423	*Aspergillus niger* (strain ATCC 1015 / CBS 113.46 / FGSC A1144 / LSHB Ac4 / NCTC 3858a / NRRL 328 / USDA 3528.7)	Cytoplasm
PKS-NRPS hybrid synthetase swnK	E9F8M3	*Metarhizium robertsii* (strain ARSEF 23 / ATCC MYA-3075)	Cytoplasm
Ceramide-binding protein SVF1	Q6CQY2	*Kluyveromyces lactis* (strain ATCC 8585 / CBS 2359 / DSM 70799 / NBRC 1267 / NRRL Y-1140 / WM37)	Golgi apparatus
Malformin synthetase mlfA	A0A317VEE1	*Aspergillus eucalypt*icola (strain CBS 122712 / IBT 29274)	Unknown

pH-response regulator protein palA/RIM20	P0CM47	*Cryptococcus neoformans* var. *neoformans* serotype D (strain B-3501A)	Unknown
**Cell wall organization**			
GPI ethanolamine phosphate transferase 1	P36051	*Saccharomyces cerevisiae* (strain ATCC 204508 / S288c)	Cell wall
Protein kinase C-like 2	P36583	*Schizosaccharomyces pombe* (strain 972 / ATCC 24843)	Unknown
**Unknown**			
Amidohydro-rel domain-containing protein	A0A2H3GRT8	*Fusarium oxysporum* f. sp. *radicis-cucumerinum*	Unknown
DDHD domain-containing protein	A0A2H3GEF8	*Fusarium oxysporum f. sp. radicis-cucumerinum*	Unknown
DRIM domain-containing protein	A0A2H3HTW8	*Fusarium oxysporum f. sp. radicis-cucumerinum*	Unknown
CBP3-like protein	Q9USK6	*Schizosaccharomyces pombe* (strain 972 / ATCC 24843)	Mitochondria

Supplementary Table S2 – Unique proteins identified in the DVL lectin-treated group by ESI-LC-MS/MS

Protein Name	ID UniProt	Organism Reference	Cellular Compartment
**Intracellular Protein Transport**			
Vacuolar protein sorting-associated protein 70	P47161	*Saccharomyces cerevisiae* (strain ATCC 204508 / S288c)	Membrane
Dynamin-like GTPase MGM1, mitochondrial	P32266	*Saccharomyces cerevisiae* (strain ATCC 204508 / S288c)	Cytoplasm
Probable GTPase-activating protein GYL1	Q04322	*Saccharomyces cerevisiae* (strain ATCC 204508 / S288c)	Cytoplasm
Vacuolar protein-sorting-associated protein 24	P36095	*Saccharomyces cerevisiae* (strain ATCC 204508 / S288c)	Cytoplasm
Coatomer subunit beta	P41810	*Saccharomyces cerevisiae* (strain ATCC 204508 / S288c)	Golgi membrane
Biogenesis of lysosome-related organelles complex 1 subunit CNL1	A0A2H3HN27	*Fusarium oxysporum* f. sp. *radicis-cucumerinum* OX=327505	Unknown
Importin N-terminal domain-containing protein	A0A2H3HGD0	*Fusarium oxysporum* f. sp. *radicis-cucumerinum* OX=327505	Cytoplasm
Golgi SNAP receptor complex member 1	P38736	*Saccharomyces cerevisiae* (strain ATCC 204508 / S288c)	Golgi membrane
Probable E3 ubiquitin-protein ligase TOM1	Q756G2	*Ashbya gossypii* (strain ATCC 10895 / CBS 109.51 / FGSC 9923 / NRRL Y-1056)	Cytoplasm
Chitin biosynthesis protein CHS6	P40955	*Saccharomyces cerevisiae* (strain ATCC 204508 / S288c)	Membrane
Probable ADP-ribosylation factor-binding protein C1F3.05	Q10410	*Schizosaccharomyces pombe* (strain 972 / ATCC 24843)	Cytosol
Autophagy-related protein 18	P0CS29	*Cryptococcus neoformans* var. *neoformans serotype* D (strain B-3501A)	Membrane
**Cell cicle**			

Serine/threonine-protein kinase RCK1	P38622	*Saccharomyces cerevisiae* (strain ATCC 204508 / S288c)	Nucleus
Kinesin-like protein CIN8	P27895	*Saccharomyces cerevisiae* (strain ATCC 204508 / S288c)	Nucleus
Pre-mRNA-splicing factor CEF1	Q03654	*Saccharomyces cerevisiae* (strain ATCC 204508 / S288c)	Cytoplasm
Inner centromere protein-related protein SLI15	P38283	*Saccharomyces cerevisiae* (strain ATCC 204508 / S288c)	Cytoplasm
Partitioning protein REP2	P03872	*Saccharomyces cerevisiae* (strain ATCC 204508 / S288c)	Nucleus
Meiosis-specific protein HOP1	P20050	*Saccharomyces cerevisiae* (strain ATCC 204508 / S288c)	Nucleus
Spindle pole body component KRE28	Q04431	*Saccharomyces cerevisiae* (strain ATCC 204508 / S288c)	Cytoplasm
Cohesin subunit psc3	O13816	*Schizosaccharomyces pombe* (strain 972 / ATCC 24843)	Nucleus
ATP-dependent DNA helicase chl1	O14147	*Schizosaccharomyces pombe* (strain 972 / ATCC 24843)	Nucleus
Cell division control protein 13	P32797	*Saccharomyces cerevisiae* (strain ATCC 204508 / S288c)	Chromosome
Spindle pole body component KRE28	A7TFD7	*Vanderwaltozyma polyspora* (strain ATCC 22028 / DSM 70294 / BCRC 21397 / CBS 2163 / NBRC 10782 / NRRL Y-8283 / UCD 57-17)	Cytoplasm
Enhancer of polycomb-like protein 1	Q6BNX0	*Debaryomyces hansenii* (strain ATCC 36239 / CBS 767 / BCRC 21394 / JCM 1990 / NBRC 0083 / IGC 2968)	Nucleus
Altered inheritance of mitochondria protein 20	B3LU06	*Saccharomyces cerevisiae* (strain RM11-1a)	Nucleus
Probable kinetochore protein NDC80	Q6BPZ9	*Debaryomyces hansenii* (strain ATCC 36239 / CBS 767 / BCRC 21394 / JCM 1990 / NBRC 0083 / IGC 2968)	Nucleus
G2/mitotic-specific cyclin cig1	P10815	*Schizosaccharomyces pombe* (strain 972 / ATCC 24843)	Chromatin
Tubulin gamma chain	P32348	*Microbotryum violaceum*	Cytoplasm
NASP-related protein sim3	Q9USQ4	*Schizosaccharomyces pombe* (strain 972 / ATCC 24843)	Chromatin
Protein STU1	P0CM75	*Cryptococcus neoformans* var. *neoformans serotype* D (strain B-3501A)	Cytoplasm

Protein biosynthesis

T-complex protein 1 subunit alpha	P12612	*Saccharomyces cerevisiae* (strain ATCC 204508 / S288c)	Plasme Membrane
Elongation factor 3B	P53978	*Saccharomyces cerevisiae* (strain ATCC 204508 / S288c)	Ribosome
Intermediate cleaving peptidase 55	P40051	*Saccharomyces cerevisiae* (strain ATCC 204508 / S288c)	Mitochondria
Deoxyhypusine synthase	Q75EW4	*Ashbya gossypii* (strain ATCC 10895 / CBS 109.51 / FGSC 9923 / NRRL Y-1056)	Cytoplasm
Eukaryotic translation initiation factor 6	B8MDN4	Talaromyces stipitatus (strain ATCC 10500 / CBS 375.48 / QM 6759 / NRRL 1006)	Cytoplasm
Probable zinc metalloprotease MCYG_04217	C5FP82	*Arthroderma otae* (strain ATCC MYA-4605 / CBS 113480)	Cytoplasm
tRNA (guanine(37)-N1)-methyltransferase	Q8SVV3	*Encephalitozoon cuniculi* (strain GB-M1)	Mitochondrial matrix
tRNA (guanine(9)-N1)-methyltransferase	Q6FQB2	*Candida glabrata* (strain ATCC 2001 / BCRC 20586 / JCM 3761 / NBRC 0622 / NRRL Y-65 / CBS 138)	Cytoplasm
Eukaryotic translation initiation factor 3 subunit B	Q5BGH1	*Emericella nidulans* (strain FGSC A4 / ATCC 38163 / CBS 112.46 / NRRL 194 / M139)	Cytoplasm
**Transferase**			
Ribosomal lysine N-methyltransferase 4	Q12504	*Saccharomyces cerevisiae* (strain ATCC 204508 / S288c)	Nucleus
Non-specific serine/threonine protein kinase	A0A2H3GY15	*Fusarium oxysporum* f. sp. *radicis-cucumerinum* OX=327505	Nucleus
tRNA(His) guanylyltransferase	Q9Y7T3	*Schizosaccharomyces pombe* (strain 972 / ATCC 24843)	Nucleus
Glycylpeptide N-tetradecanoyltransferase	Q9UVX3	*Aspergillus fumigatu*s (strain ATCC MYA-4609 / CBS 101355 / FGSC A1100 / Af293)	Cytoplasm
**Stress and Defense Response**			
SVF1-like protein YDR222W	Q04925	*Saccharomyces cerevisiae* (strain ATCC 204508 / S288c)	Nucleus
Catalase domain-containing protein	A0A2H3HAL5	*Fusarium oxysporum* f. sp. *radicis-cucumerinum* OX=327505	Cytoplasm
SVP1-like protein 2	P50079	*Saccharomyces cerevisiae* (strain ATCC 204508 / S288c)	Cytoplasm

Protein FYV8	Q6FSM8	*Candida glabrata* (strain ATCC 2001 / BCRC 20586 / JCM 3761 / NBRC 0622 / NRRL Y-65 / CBS 138)	Unknown
**Gene regulation**			
S-methyl-5'-thioadenosine phosphorylase	Q07938	*Saccharomyces cerevisiae* (strain ATCC 204508 / S288c)	Cytoplasm
Zn(2)-C6 fungal-type domain-containing protein	A0A2H3H7V0	*Fusarium oxysporum* f. sp. *radicis-cucumerinum* OX=327505	Nucleus
**Metabolism and synthesis of amino acids**			
Uroporphyrinogen-III C-methyltransferase	P36150	*Saccharomyces cerevisiae* (strain ATCC 204508 / S288c)	Nucleus
Pentafunctional AROM polypeptide	C5DN02	*Lachancea thermotolerans* (strain ATCC 56472 / CBS 6340 / NRRL Y-8284)	Cytoplasm
**DNA repair**			
DNA mismatch repair protein MSH2	P25847	*Saccharomyces cerevisiae* (strain ATCC 204508 / S288c)	Nucleus
ATP-dependent DNA helicase	A0A2H3G8E2	*Fusarium oxysporum* f. sp. *radicis-cucumerinum* OX=327505	Nucleus
ATP-dependent DNA helicase II subunit 2	Q7RX73	*Neurospora crassa* (strain ATCC 24698 / 74-OR23-1A / CBS 708.71 / DSM 1257 / FGSC 987)	Chromosome
General transcription and DNA repair factor IIH subunit TFB4	Q6BL86	*Debaryomyces hansenii* (strain ATCC 36239 / CBS 767 / BCRC 21394 / JCM 1990 / NBRC 0083 / IGC 2968)	Nucleus
**Transcription regulation**			
Glucose transport transcription regulator RGT1	P32862	*Saccharomyces cerevisiae* (strain ATCC 204508 / S288c)	Cytoplasm
Mediator of RNA polymerase II transcription subunit 13	A0A2H3GQW6	*Fusarium oxysporum* f. sp. *radicis-cucumerinum* OX=327505	Cytoplasm
High-osmolarity-induced transcription protein 1	Q755E7	*Ashbya gossypii* (strain ATCC 10895 / CBS 109.51 / FGSC 9923 / NRRL Y-1056)	Nucleus
Mating-type protein MAT-1	O13402	*Cochliobolus carbonum* (strain 26-R-13)	Nucleus

Mating type protein SmtA-1	O42837	*Sordaria macrospora* (strain ATCC MYA-333 / DSM 997 / K(L3346) / K-hell)	Nucleus
26S proteasome regulatory subunit RPN1	Q03465	*Saccharomyces cerevisiae* (strain ATCC 204508 / S288c)	Cytoplasm
Glucose transport transcription regulator RGT1	B3LR49	*Saccharomyces cerevisiae* (strain RM11-1a)	Cytoplasm
Transcription factor tau 131 kDa subunit	P33339	*Saccharomyces cerevisiae* (strain ATCC 204508 / S288c)	Cytoplasm
Hps1-dma1 cluster transcription factor tfc7	M2XJV1	*Dothistroma septosporum* (strain NZE10 / CBS 128990)	Nucleus
**Regulation Factor and RNA Processing**			
rDNA transcriptional regulator POL5	P39985	*Saccharomyces cerevisiae* (strain ATCC 204508 / S288c)	Nucleolus
tRNA N6-adenosine threonylcarbamoyltransferase	P36132	*Saccharomyces cerevisiae* (strain ATCC 204508 / S288c)	Cytoplasm
ATP-dependent RNA helicase DBP10	A0A2H3HQ48	*Fusarium oxysporum* f. sp. *radicis-cucumerinum* OX=327505	Nucleolus
PAD domain-containing protein	A0A2H3GHV8	*Fusarium oxysporum* f. sp. *radicis-cucumerinum* OX=327505	Cytoplasm
ATP-dependent RNA helicase MAK5	A5DPU0	*Meyerozyma guilliermondii* (strain ATCC 6260 / CBS 566 / DSM 6381 / JCM 1539 / NBRC 10279 / NRRL Y-324)	Nucleolus
Enhancer of mRNA-decapping protein 3	O94752	*Schizosaccharomyces pombe* (strain 972 / ATCC 24843)	Cytoplasm
Putative tRNA 2'-phosphotransferase	O14045	*Schizosaccharomyces pombe* (strain 972 / ATCC 24843)	Nucleus
Endoribonuclease YSH1	Q4PEJ3	*Ustilago maydis* (strain 521 / FGSC 9021)	Nucleus
ATP-dependent rRNA helicase SPB4	Q0UP45	*Phaeosphaeria nodorum* (strain SN15 / ATCC MYA-4574 / FGSC 10173)	Nucleolus

Cytoplasmic tRNA 2-thiolation protein 2	Q6CF50	*Yarrowia lipolytica* (strain CLIB 122 / E 150)	Cytoplasm
**Carbohydrate metabolism**			
6-phosphogluconolactonase-like protein 1	P50278	*Saccharomyces cerevisiae* (strain ATCC 204508 / S288c)	Cytoplasm
Galactose-1-phosphate uridylyltransferase	P08431	*Saccharomyces cerevisiae* (strain ATCC 204508 / S288c)	Cytoplasm
Mannan endo-1,6-alpha-mannosidase	A0A2H3G P70	*Fusarium oxysporum* f. sp. *radicis-cucumerinum* OX=327505	Membrane
Beta-hexosaminidase	P43077	*Candida albicans*	Membrane
Probable pectin lyase A	Q4WV10	Aspergillus fumigatus (strain ATCC MYA-4609 / CBS 101355 / FGSC A1100 / Af293)	Extracellular region
Probable glucan endo-1,3-beta-glucosidase btgC	Q0CI96	*Aspergillus terreus* (strain NIH 2624 / FGSC A1156)	Plasme membrane
Putative rhamnogalacturonase D	A2QWT2	*Aspergillus niger* (strain ATCC MYA-4892 / CBS 513.88 / FGSC A1513)	Extracellular region
**Transmembrane transporters**			
General amino-acid permease GAP1	P19145	*Saccharomyces cerevisiae* (strain ATCC 204508 / S288c)	Endossome
ATP-dependent bile acid permease	P32386	*Saccharomyces cerevisiae* (strain ATCC 204508 / S288c)	Endoplasmic reticulum
Azole resistance protein 1	P50080	*Saccharomyces cerevisiae* (strain ATCC 204508 / S288c)	Plasm membrane
MFS domain-containing protein	A0A2H3H NM3	*Fusarium oxysporum* f. sp. *radicis-cucumerinum* OX=327505	Membrane
Cycloheximide resistance protein	P32071	*Candida maltosa*	Membrane
MFS-type transporter acdC	A0A2I2F27 1	*Aspergillus candidus*	Membrane
Protein ZDS1	P50111	*Saccharomyces cerevisiae* (strain ATCC 204508 / S288c)	Cytoplasm

Probable high-affinity hexose transporter ght8, mitochondrial	Q9P3U7	*Schizosaccharomyces pombe* (strain 972 / ATCC 24843)	Mitochondrial Membrane
Probable NADPH:adrenodoxin oxidoreductase	P48360	*Saccharomyces cerevisiae* (strain ATCC 204508 / S288c)	Mitochondria
**Lipid biosynthesis**			
Fatty acid synthase subunit alpha	P19097	*Saccharomyces cerevisiae* (strain ATCC 204508 / S288c)	Mitochondria
Naringenin synthase	W3X8X8	*Pestalotiopsis fici* (strain W106-1 / CGMCC3.15140)	Cytoplasm
Hybrid PKS-NRPS synthetase ATEG_0032	Q0D159	*Aspergillus terreus* (strain NIH 2624 / FGSC A1156)	Cytoplasm
Elongation of fatty acids protein 1	P39540	*Saccharomyces cerevisiae* (strain ATCC 204508 / S288c)	Endoplasmic reticulum
Hybrid PKS-NRPS synthetase iliA	P0DO30	*Neonectria* sp. (strain DH2)	Cytoplasm
Hybrid PKS-NRPS synthetase traA	A0A481WNP4	*Penicillium crustosum*	Unknown
**Sporulation**			
Negative regulator of sporulation PMD1	P32634	*Saccharomyces cerevisiae* (strain ATCC 204508 / S288c)	Cytoplasm
Sporulation-specific protein 22	P40511	*Saccharomyces cerevisiae* (strain ATCC 204508 / S288c)	Nucleus
**Nucleotide metabolism**			
Y' element ATP-dependent helicase YML133C	Q03099	*Saccharomyces cerevisiae* (strain ATCC 204508 / S288c)	Cytoplasm
Adenosine kinase	P47143	*Saccharomyces cerevisiae* (strain ATCC 204508 / S288c)	Cytoplasm
m7GpppX diphosphatase	Q06151	*Saccharomyces cerevisiae* (strain ATCC 204508 / S288c)	Cytoplasm
Nicotinate-nucleotide pyrophosphorylase [carboxylating]	A0A2H3HTR1	*Fusarium oxysporum* f. sp. *radicis-cucumerinum* OX=327505	Nucleus
**Cell wall organization**			
Chitin synthase 1	P08004	*Saccharomyces cerevisiae* (strain ATCC 204508 / S288c)	Plasm membrane
Cell wall mannoprotein PIR3	Q03180	*Saccharomyces cerevisiae* (strain ATCC 204508 / S288c)	Cell wall

Chitin synthase	A0A2H3HHV3	*Fusarium oxysporum* f. sp. *radicis-cucumerinum* OX=327505	Plasm membrane
**Metabolism and energy**			
Flavin prenyltransferase PAD1, mitochondrial	A0A2H3HNG2	*Fusarium oxysporum* f. sp. *radicis-cucumerinum* OX=327505	Mitochondria
Amino_oxidase domain-containing protein	A0A2H3GTT9	*Fusarium oxysporum* f. sp. *radicis-cucumerinum* OX=327505	Unknown
Alkaline phosphatase	A0A2H3HHY5	*Fusarium oxysporum* f. sp. *radicis-cucumerinum* OX=327505	Unknown
AB hydrolase-1 domain-containing protein	A0A2H3I1B9	*Fusarium oxysporum* f. sp. *radicis-cucumerinum* OX=327505	Cytoplasm
Peptidase_S8 domain-containing protein	A0A2H3G634	*Fusarium oxysporum* f. sp. *radicis-cucumerinum* OX=327505	Cytoplasm
SURF1-like protein	A0A2H3GS11	*Fusarium oxysporum* f. sp. *radicis-cucumerinum* OX=327505	Mitochondrial Inner Membrane
Malformin synthetase mlfA	A0A319DV72	*Aspergillus sclerotiicarbonarius* (strain CBS 121057 / IBT 28362)	Cytoplasm
Glyceraldehyde-3-phosphate dehydrogenase	P87197	*Hypocrea atroviridis*	Cytoplasm
Prenyltransferase asqH1	C8VJQ1	*Emericella nidulans* (strain FGSC A4 / ATCC 38163 / CBS 112.46 / NRRL 194 / M139)	Cytoplasm
Bifunctional lycopene cyclase/phytoene synthase	S0EQ07	*Gibberella fujikuroi* (strain CBS 195.34 / IMI 58289 / NRRL A-6831)	Membrane
Cytochrome P450 monooxygenase astA	A0A3Q9FEJ4	*Talaromyces wortmannii*	Membrane
Nonribosomal peptide synthetase sidC	Q4WR82	*Aspergillus fumigatus* (strain ATCC MYA-4609 / CBS 101355 / FGSC A1100 / Af293)	Cytoplasm
NADP-specific glutamate dehydrogenase	Q9HFR6	*Tuber borchii*	Unknown
**Intracellular regulation**			

F-box domain-containing protein	A0A2H3GTI6	*Fusarium oxysporum* f. sp. *radicis-cucumerinum* OX=327505	Cytoplasm
Inner nuclear membrane protein HEH2	Q03281	*Saccharomyces cerevisiae* (strain ATCC 204508 / S288c)	Nucleus
F-box protein pof9	O74381	*Schizosaccharomyces pombe* (strain 972 / ATCC 24843)	Cytoplasm
Vacuolar membrane-associated protein iml1	A1DFV9	*Neosartorya fischeri* (strain ATCC 1020 / DSM 3700 / CBS 544.65 / FGSC A1164 / JCM 1740 / NRRL 181 / WB 181)	Vacuolar membrane
**Oxireductase**			
Aldedh domain-containing protein	A0A2H3G7X6	*Fusarium oxysporum* f. sp. *radicis-cucumerinum* OX=327505	Unknown
**Unknown**			
EthD domain-containing protein	A0A2H3GI75	*Fusarium oxysporum* f. sp. *radicis-cucumerinum* OX=327505	Unknown
NACHT domain-containing protein	A0A2H3G3U7	*Fusarium oxysporum* f. sp. *radicis-cucumerinum* OX=327505	Unknown
Methyltransf_33 domain-containing protein	A0A2H3FQB7	*Fusarium oxysporum* f. sp. *radicis-cucumerinum* OX=327505	Unknown
zinc_ribbon_16 domain-containing protein	A0A2H3HMR8	*Fusarium oxysporum* f. sp. *radicis-cucumerinum* OX=327505	Unknown
Goodbye domain-containing protein	A0A2H3GBL2	*Fusarium oxysporum* f. sp. *radicis-cucumerinum* OX=327505	Unknown
Epimerase domain-containing protein	A0A2H3GTA7	*Fusarium oxysporum* f. sp. *radicis-cucumerinum* OX=327505	Unknown
Aldo_ket_red domain-containing protein	A0A2H3GZR4	*Fusarium oxysporum* f. sp. *radicis-cucumerinum* OX=327505	Unknown
Abhydrolase_3 domain-containing protein	A0A2H3GF32	*Fusarium oxysporum* f. sp. *radicis-cucumerinum* OX=327505	Unknown
RING-type domain-containing protein	A0A2H3HT78	*Fusarium oxysporum* f. sp. *radicis-cucumerinum* OX=327505	Unknown

Fungal_trans domain-containing protein	A0A2H3GV43	*Fusarium oxysporum* f. sp. *radicis-cucumerinum* OX=327505	Nucleus
